# Multi-scaled Monte Carlo calculation for radon-induced cellular damage in the bronchial airway epithelium

**DOI:** 10.1038/s41598-021-89689-0

**Published:** 2021-05-13

**Authors:** Ali Abu Shqair, Eun-Hee Kim

**Affiliations:** Department of Nuclear Engineering, Seoul National University, Seoul, 08826 Republic of Korea

**Keywords:** Biophysics, Computational biophysics, Nanoscale biophysics

## Abstract

Radon is a leading cause of lung cancer in indoor public and mining workers. Inhaled radon progeny releases alpha particles, which can damage cells in the airway epithelium. The extent and complexity of cellular damage vary depending on the alpha particle’s kinetic energy and cell characteristics. We developed a framework to quantitate the cellular damage on the nanometer and micrometer scales at different intensities of exposure to radon progenies Po-218 and Po-214. Energy depositions along the tracks of alpha particles that were slowing down were simulated on a nanometer scale using the Monte Carlo code Geant4-DNA. The nano-scaled track histories in a 5 μm radius and 1 μm-thick cylindrical volume were integrated into the tracking scheme of alpha trajectories in a micron-scale bronchial epithelium segment in the user-written SNU-CDS program. Damage distribution in cellular DNA was estimated for six cell types in the epithelium. Deep-sited cell nuclei in the epithelium would have less chance of being hit, but DNA damage from a single hit would be more serious, because low-energy alpha particles of high LET would hit the nuclei. The greater damage in deep-sited nuclei was due to the 7.69 MeV alpha particles emitted from Po-214. From daily work under 1 WL of radon concentration, basal cells would respond with the highest portion of complex DSBs among the suspected progenitor cells in the most exposed regions of the lung epithelium.

## Introduction

Radon is a gaseous radioactive nuclide that transforms to radioactive progeny with short half-lives and that emits alpha particles. Alpha particles that have high linear energy transfer (LET) emitted from the progeny deposited on the mucous layer can damage cells in the bronchial airway, which possibly leads to lung cancer. The relationship between radon exposure and cancer has been demonstrated^1,2^. Exposure from inhaled radon gas and its progeny represent approximately 50% of the annual public exposure^3^, and 3–14% of the total lung cancers are attributed to radon exposure^4^. Experimental^5–8^ and theoretical^9,10^ studies evidenced that radon progeny particles are heterogeneously deposited on the surface of airway epithelium. High concentration of particle deposition was experimentally observed in the large bronchial region^11,12^. Computational fluid dynamics studies showed substantial depositions of radon progeny at the carinal ridge^9,13,14^.

The impact of radon and progeny inhalation on lung cells has been investigated on a micron-scale by theoretical analyses. Cellular death^15–18^ and mutation probability^18^ are proportional to the number of alpha-particle traversals in the cell nuclei according to various experiments. Balashazy et al.^9^ showed that the heterogeneous deposition of radon progeny in the airway bifurcation leads to multiple hits among cells in hot spots, and the dose that the nucleus absorbed at hot spots reaches 22 Gy; the average nucleus dose is approximately 0.1 Gy. The probability of multiple hits at hot spots can be underestimated assuming uniform deposition of radon progeny. The heterogeneity of deposition can be mediated by mucociliary clearance. Farkas^19^ simulations showed that a mucociliary clearance is slow in the carinal region compared with other regions but the deposit is reduced to a third. Madas and Balashazy^20^ estimated transformations at different activities of radon progeny on the hot spots and suggested a threshold of dose rate over which tissue regeneration cannot recoup radiation-induced cellular death. Farkas et al.^21^ suggested the significance of the spatial and temporal patterns of cell hits in terms of biological effect and showed that lung cells in the carinal ridge can receive multiple hits within one cell cycle, and hit cells are close to one another.

Cells of the airway epithelium vary in cellular and nuclear volumes, frequency, and depth-distribution within the epithelium. They can be categorized into six types, namely, pre-ciliated, ciliated, goblet, basal, indetermined, and secretory cells^22^. The chance of being hit by an alpha particle emitted from the mucous layer varies depending on the depth of the cell’s location in the epithelium. The energy distribution of alpha particles hitting cells varies with the cell depth and thus the average energy deposition to the cells differs with the cell depth. Various geometrical aspects of cells lead to a varying distribution of particle track length in the cell and cell nucleus. Basal and secretory cells are considered to be responsible for radon-induced lung cancer^23^. Basal cells, which are the progenitors of ciliated and goblet cells^24^, are suspected due to their ability to divide and differentiate. Secretory cells also have the ability of division and differentiation to basal or ciliated cells^25^.

Energy depositions cascade in cell nuclei along the trajectory of the slowing down alpha particles, which can cause single-strand break (SSB) or double-strand breaks (DSBs). DSBs consisting of two or more SSBs on separate strands within 10 base pair distance can be repaired correctly or can lead to mutations either directly or indirectly^26,27^. The yield of DSB per absorbed dose in the nucleus varies depending on the kinetic energy of alpha particles^28,29^ and the DNA density in nucleus^30^. Generally, DSB that consists of two SSBs is called a simple DSB. A complex DSB consists of more than two SSBs. Nikjoo et al.^31^ showed that the proportion of complex DSBs increases with increasing LET level of particulate radiation. Several mechanistic models suggested that complex DSBs are repaired more slowly than simple DSBs^32–34^. An additional level of damage complexity attributes to the production of multiple DSBs in proximity. Induction of complex DSBs or proximal DSBs in the genome structure implies increased damage effect in terms of cellular death and mutation due to delayed and less efficient repair compared with simple DSBs^35–37^.

The key issue in this work is to perform nano-scale tracking of alpha particles. Nano-scale tracking is necessary for simulating the interaction of alpha particles and DNAs because (1) DNAs, the critical target, are nanoscale entities and (2) alpha particles deliver energy of a large amount in discrete locations. DNA damage of the cells in the lung epithelium is the issue relating to the cancer risk from natural radiation exposure^38^. Various simulation tools, such as Geant4-DNA^39^ and PARTRAC^40^, enable the modelling of energy depositions in cell nuclei and their interactions on the nanometer scale with DNA molecules. Unfortunately, such codes encompass in the algorithms all the details including nucleus genome and chemical processes, thereby making large-scale computations infeasible. We developed a computational scheme that enables estimating the nanometer-scaled DNA damage of cells in the micrometer-scaled lung epithelium caused by alpha-particle emissions from the radon progeny depositions on the epithelium at a feasible computational cost.

## Methods

We utilized the Geant4-DNA to simulate the nanometer-scaled energy deposition by alpha particles per unit track length in liquid water medium. We counted the DSB clusters and complex DSBs by using the Density-Based Spatial Clustering of Applications with Noise (DBSCAN) algorithm^41,42^. Nanometer-scaled DNA damage estimates were integrated in our new program to calculate DNA damages in micrometer-scaled cell nuclei of varying sizes. The Geant4 Livermore physics scheme was utilized to simulate the alpha tracks in a micrometer-scaled bronchial epithelium segment and record the sequences of cellular hits by alpha particles of varying energies. DNA damages in lung epithelial cells were evaluated for exposure to radon progeny of varying activities residing in the luminal surface of the epithelium.

### Nano-scaled simulation of alpha-particle track structure

The Geant4 toolkit^43^ that includes the Geant4-DNA default physics processes^44^ was used to simulate the alpha-particle track structure in a unit volume of 5 μm radius and 1 μm-thick cylinder. This volume represents 1 μm-thick segment of cell nucleus. In Geant4-DNA physics, an alpha particle and secondary electrons are tracked down for every event of excitation, ionization, elastic scattering, and charge transfer of alpha particle^45,46^. Alpha particles carrying initial energy ranging from 8 MeV to 10 keV were directed toward the cylindrical unit volume of water in a direction parallel to the axis. For each energy, $10^{4}$ independent track histories were generated. Each track history was characterized by the spatial distribution of interaction events and energy depositions.

At every energy deposition spot in the DNA strand, whether or not an SSB occurs was determined by the deposited energy-dependent damage induction probability^47^, which was zero for energy deposition at below 5 eV and linearly increased with the energy deposition until a value of 1 for 37.5 eV of energy deposition was reached. The linearity of DNA damage production with energy deposition was proposed earlier by Friedland et al.^47^ and adopted in PARTRAC code^48^.

A damaged strand from a helical double-strand was selected randomly by equal chances. The number of DSB clusters was calculated through the DBSCAN algorithm, which finds the clusters formed by at least two SSBs in opposite strands within a 10 base pair (3.3 nm) in radial distance. Additionally, DSB clusters consisting of more than two SSBs were reserved as complex DSB clusters.

The Geant4-DNA simulation proceeded by assuming that 20% of energy depositions from alpha particle and the secondary electrons occurred in DNA strands, and 80% occurred in the surrounding volume without indirect actions. Bernal et al.^46^ stated that the Geant4-DNA simulation based on those assumptions gave the ratio of DSB/SSB in good approximation to the value obtained from PARTRAC simulation that used a detailed genome structure and counted indirect actions.

### Consideration of the size of the cell nucleus

The scheme of quantifying the DNA damage by a single alpha track in a unit cylindrical volume of 5 µm in radius and 1 µm in depth can be applied to 1 µm of unit track length in different cell nuclei by scaling the estimate of DSB cluster with the volume fraction of chromatin fiber in each type of cell nuclei. The compaction properties of chromatin fiber in human cell nuclei were extracted from the PARTRAC code^40^. For human cell nuclei, it is reasonably assumed that chromatin fibers have the same compaction of genome content. Assuming that a cylindrical shape of chromatin fiber is randomly distributed in the nucleus, the volume fraction of chromatin fiber in the nucleus $(p_{F} )$ is calculated as follows:

$$p_{F} = { }\frac{{total\,chromatin\,fiber\,volume}}{{Nucleus\,volume}} = \frac{{\pi {R_{Fiber}^{2}} \left( {Chromatin\,fiber\,length} \right)}}{{4\pi /3\left( {R_{nucleus} } \right)^{3} }}.$$

The quantity of “potential” DNA damage obtained from the Geant4-DNA simulation would be scaled by $p_{F}$ to give the quantity of DNA damage in a 1 μm-thick segment of the specific cell nucleus.

### SNU-code for simulating cellular damage from a single alpha track

This code was designated as the “Seoul National University code for Cellular Damage Simulation” (SNU-CDS). Its goal is a fast large-scale assessment of cellular damage for the cells of varying nucleus volumes. This code simulates alpha trajectories in a micron-scaled target and computes the nanoscale quantities of cellular damage by using the alpha trajectory histories collected by running the Geant4-DNA in the unit cylindrical volume. Individual histories of alpha trajectory were specified by the energy of an alpha particle entering the unit cylinder, the corresponding LET, total energy depositions, the number of potential DSB clusters, and the number of potential complex DSBs.

**Figure 1:**
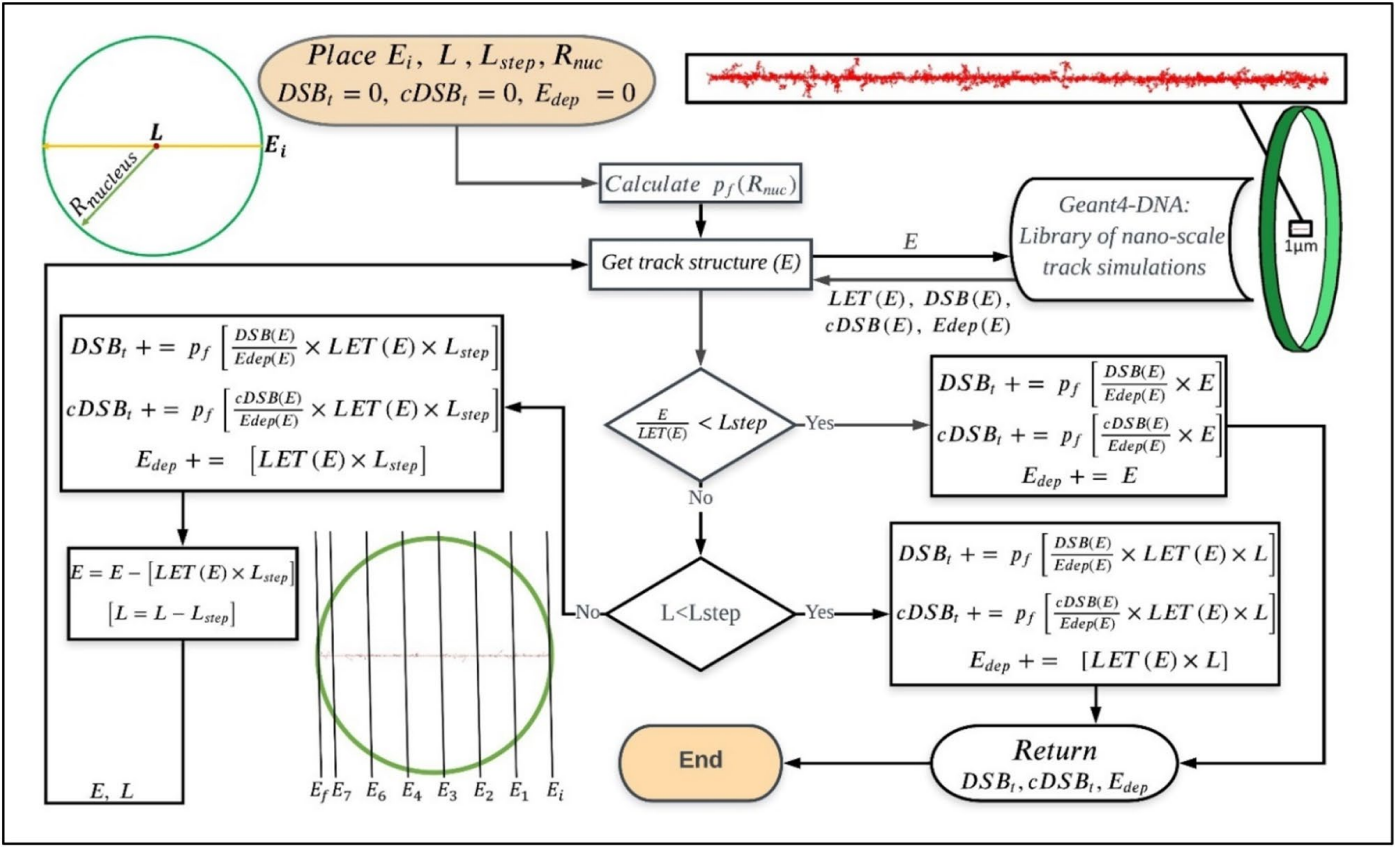
Flowchart of the SNU-CDS code. The code runs while an alpha particle carrying energy **E** penetrates a cell nucleus of radius **R**_nuc_ and completes its track of a chord length **L** in the nucleus with the maximum step length = **L**_step_. SNU-CDS code computes the total energy depositions, number of DSB, and complex DSBs in the nucleus by using the alpha trajectory histories generated by running the Geant4-DNA in a unit cylindrical volume.

The DNA damage distribution per unit track length generated in the previous step was reserved as a library for use in micron-scale simulations. For an alpha particle coming into a cellular target or a nucleus, DNA damage is assessed by numerical integration of the library data, which corresponded to the kinetic energy on every event spot of the alpha particle and its chord length in the nucleus volume. Figure 1 shows the flowchart of SNU-CDS computation. In each step ($L_{step}$) along the chord length, the energy deposition and DNA damage yields were calculated from the damage distribution specific to the energy of the alpha particle at the start of the step. The DNA damage obtained was then scaled by the cell type-specific volume fraction $p_{F}$. For the energy between the simulated discrete energies of the alpha particle, the energy deposition and DNA damage yields were determined through linear interpolation.

**Figure 2:**
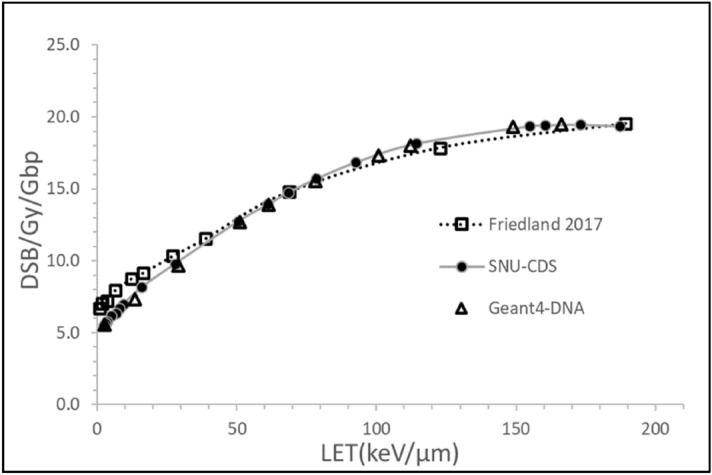
DSB yields per nucleus dose computed for a 10 μm diameter spherical model using the SNU-CDS algorithm (filled circles) and the Geant4-DNA (triangles) as a function of LET corresponding to the alpha-particle energy and in comparison with the estimates of Friedland et al. (squares)^28^.

The alpha particle continues generating discrete events until the end of chord length or when it comes to rest. The SNU-CDS scheme was validated by comparing its estimates of DSB yields per nucleus dose with the estimates obtained by the Geant4-DNA simulation over a full scale of nucleus volume. The scheme was tested by application to the model of Friedland et al.^28^. These three estimates for a 10 μm diameter cell nucleus are shown in Fig. 2. The value of DNA fiber radius ($R_{Fiber}^{ }$) was tuned to the PARTRAC model, which assumes the genome size of 6.6 Gbp and the compaction of 5.56 kbp in a 50 nm block of chromatin fiber, to obtain the results. The total length of chromatin fiber in a nucleus was 59.35 mm. The comparable DSB/Gy/Gbp to the estimate from PARTRAC could be expected when the radius of chromatin fiber was approximately 16 nm.

### Integration of cellular damage over isotropic crossings of alpha tracks

Earlier studies focused on the nucleus hit as a precondition of cell death or mutation^15–17^; hit probability was used to evaluate the risk of radon exposure^49–51^. We calculated the hit effectiveness in terms of average cellular damage per hit for an alpha particle carrying an energy of up to 8 MeV for the nuclei of cells constituting the lung epithelium by using the SNU-CDS code.

Experimental nucleus volume data for ciliated, goblet, basal, and secretory cells are available in Mercer et al.^22^. Pre-ciliated cells were assumed to have the same nucleus volume as ciliated cells according to Madas^52^. The nucleus volume of indetermined cells was calculated from the volumetric portion of indetermined nuclei in the large bronchial epithelium divided by the cell frequency, in accordance with the suggestion of Madas^52^ that indetermined cells have the same ratio of nucleus to cytoplasm as basal cells. Six cell types in the lung epithelium were characterized by the nominal diameters in Table 1.

**Table 1:** Nominal nucleus sizes and frequencies of six cell types in the bronchial epithelium^22^.

Cell type	Ciliated	Pre-ciliated	Goblet	Basal	Secretory	Indetermined
Nucleus volume (μm^3^)	310	310	243	201	230	156
Area cell frequency (/mm^2^)	17,900	2200	5300	17,100	1800	9600

For each alpha-particle energy, cellular damage parameters per nucleus hit were averaged over an isotropic flux of mono-energetic alpha particles hitting the nucleus surface. The distributions of cellular damage per nucleus hit were obtained by using the probability distribution of cellular damage $v\left( s \right)$, which was given as a function of the particle’s chord length ($s$) in a nucleus sphere. The cellular damage per nucleus hit averaged over all the possible angles ($\theta$)^53^ was calculated by integrating over the polar angle θ of up to π/2 to the inward radial direction of the sphere with a discrete angle span of $\Delta \theta = \frac{{\left( {\pi /2} \right)}}{1000}$.

$$\overline{V} = \frac{{\mathop \smallint \nolimits_{0}^{\pi /2} 2\pi v\left( s \right)\sin \theta \cos \theta d\theta }}{{\mathop \smallint \nolimits_{0}^{\pi /2} 2\pi \sin \theta \cos \theta d\theta }}.$$

At each $\theta$, the chord length *L* was given by *2R *cos *θ*. Energy depositions and DNA damages along the chord length *L* were calculated in the SNU-CDS algorithm with *L*_*step*_ = 10 nm. The average deposited energy and average number of DSBs and complex DSBs per nucleus hit were assessed by numerically integrating the cellular damage over the angle of *θ* up to π/2.

$$\overline{V} = \mathop \sum \limits_{0}^{1000} 2 v\left( s \right)\sin \theta \cos \theta \Delta \theta .$$

The integration of the isotropic crossing of alpha particles in this section was intended to estimate with high confidence the mean values for individual cells. In the following section, random directions of alpha particles emitting from the mucous layer and entering cells of interest, and corresponding chord lengths are considered.

### Bronchial epithelium model

**Figure 3:**
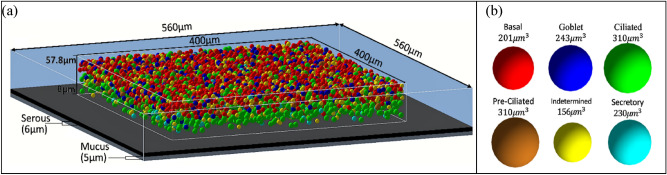
Dimensions of (**a**) the bronchial epithelium section for the simulation of alpha-particle emissions from radon progeny within the mucous layer and (**b**) six cell types constituting the epithelium section.

Figure 3a shows the simulation model of bronchial epithelium. Six types of cells, as shown in Fig. 3b, are distributed in the tissue segment. Tissue geometry modelling in this study was in line with that in previous studies^20,52^; the experimental data measured by Mercer et al.^22^ and Mercer et al.^54^ were used. A small segment (400 µm × 400 µm in area and 57.8 µm in thickness) of bronchial epithelium was filled with cell nuclei. Additional 6 µm-thick cilia (serous) and 5 µm-thick mucous layers were attached to the luminal surface^55^. Liquid water composition was used for the epithelium section at a density of 1.0 g/cm^3^ for cell nuclei and the serous and mucous layers and at a density of 1.05 g/cm^3^ for the remaining volume^56^. This segment represents the epithelium exposed to radon progeny in the airway carinal ridge. The nuclei of six cell types, including pre-ciliated, ciliated, goblet, basal, indetermined, and secretory cells, were considered as target bodies. The 5 μm-thick mucous layer was the source reservoir. Radon progeny were assumed to be uniformly mixed within the mucous layer, in accordance with the ICRU Report 88^23^.

The 57.8 μm-thick tissue segment was divided into five regions, namely, four regions with 12 μm-thick layers and one region with 9.8 μm-thick layers from the luminal surface to the basement membrane. Each region was characterized by the frequencies of individual cell types, which were calculated by using the frequencies of lung cells per unit area of the basement membrane in the human large bronchi (Table 1) as reported by Mercer et al.^22^ and the relative portions in volume of individual cell nuclei in the region. Mercer et al.^54^ provided the areal portions of six cell types in Table 2 at six discrete depths of the bronchial epithelium. The areal portions were assumed to change linearly along the discrete depths. The continuous functions of areal portions were used to define the probability density function of each cell type in each region and to calculate the volume portions over the five regions. The nuclei of each cell type were located over the depth in each region according to the chance that was proportional to its areal portion. The continuity of the probability density function for each cell type was ensured in all regions by limiting the nuclear center within each region and allowing the nuclear volume to enter the adjacent regions. The nuclear centers did not overlap and did not reside on the boundary of the tissue segment.

**Table 2:** Nucleus areal portions (%) for six cell types at varying depths from the surface of the bronchial epithelium^54^.

Cell type (μm)	Ciliated	Pre-ciliated	Goblet	Basal	Secretory	Indetermined
0	0	0	0	0	0	0
12	5	0	0	0	1.8	0.8
24	19	2.4	1	0.2	2.4	1.9
36	11	2.6	3	8.3	1.4	3.4
48	5	2	3.5	21	0.4	4.8
57.8	0	0	0	0	0	0

### Simulation of cellular damage in lung epithelium from exposure to radon progeny

**Figure 4:**
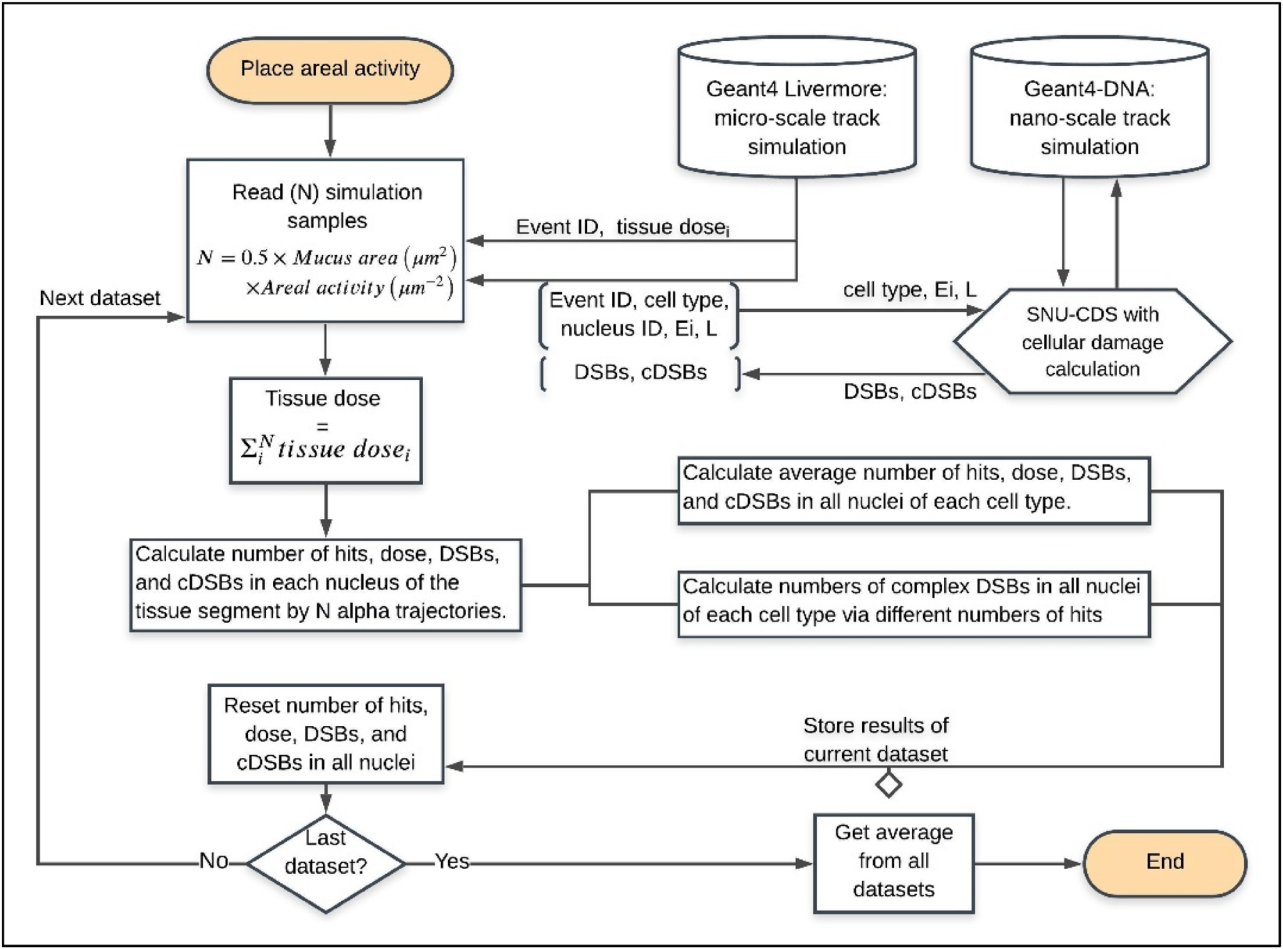
Flowchart of the C++ code, which integrates the nanometer-scaled Geant4-DNA simulation data into the micrometer-scaled Geant4 Livermore scheme to estimate the DNA damage of cells in the bronchial epithelium.

A C++ code shown in Fig. 4 was written to integrate the nanometer-scaled simulation data of DNA damage occurring along an alpha track into the micrometer-scaled simulation for damage estimation of individual cell types in the bronchial epithelium segment.

Beta and gamma emissions from the decay chain of radon progenies were neglected in our study because alpha emissions of high-LET dominate in biological impact on cells. Alpha particles were emitted isotropically from random locations within the mucous layer in the luminal side of the tissue segment. The mucous layer was expanded to have an additional 80 µm of dimension in the x–y plane, thereby ensuring the uniform flux of alpha particles at the entrance of the first layer in the tissue segment. Six cell types of different nucleus sizes (Table 1) were distributed according to the areal portions, which vary with the depth. Each alpha-particle emission was assigned with the energy from either of the radon progeny, namely, ${}_{ }^{214} {\text{Po}}$ (E = 7.69 MeV) and ${}_{ }^{218} {\text{Po}}$ (E = 6 MeV), by the chance in proportion to the activity ratio. In our simulation, the ratios of 0.896 and 0.104 were assigned to the decays of ${}_{ }^{214} {\text{Po}}$ and ${}_{ }^{218} {\text{Po}}$, respectively. These ratios characterized the highest exposed area in the carinal ridge of a worker’s lung under uranium mine exposure conditions, as reported by Madas and Balashazy^20^ and Szoke et al.^57^.

Considering that a large interaction volume and a large number of samples were required in this part, the condensed-history scheme of Geant4 Livermore physics was used by using 100 eV as an energy cutoff for electrons. By including the SNU-CDS algorithm, the condensed Livermore physics scheme would provide good approximations of the histories of alpha trajectories for nanometer-scaled targets residing in micron-scale volume to the results from the Geant4-DNA simulation^58^. The alpha energy upon entering the nucleus and the chord length in the nucleus would determine the energy deposition to the nucleus and the numbers of DBS clusters and complex DSBs formed in the nucleus. The SNU-CDS code computes the number of DSB clusters and the number of complex DSBs for each cell type by integrating DNA damage data from the track library of Geant4-DNA simulation and by applying the cell type-specific volume fraction of chromatin fiber $p_{F}$. In this way, $2 \times 10^{7}$ alpha trajectories were simulated with less computations than those in the default Geant4-DNA simulation.

Each of the $2 \times 10^{7}$ alpha trajectories resulted in a varying record for the number of DSB clusters and the number of complex DSBs for each cell type. These $2 \times 10^{7}$ records are used to compute the overall DNA damages in each cell type that varies with the amount of radon progeny depositions in the mucous layer of lung epithelium. The density of alpha emissions was correlated with exposure to inhaled radon and corresponded to the number of alpha-particle emissions from radon progeny distributed in the mucous layer. DNA damage of the cells in the lung tissue increased with the density of alpha-emissions from radon progeny in the mucous layer. The alpha-emissions ranging from 0.01 to 0.5 from a 5 µm^3^ ($1\,\upmu{\text{m}} \times 1\, \upmu{\text{m}} \times 5\,\upmu{\text{m}}$) section of the mucous layer were chosen to reflect the real working environment. The concentration of alpha decays reached 0.71 µm^−2^ at the most exposed surface of the bronchial epithelium after 8 h of work in the mine^14,20^. From the estimates for varying alpha-emission densities, more variation in damage was found in low emission density than in high density regardless of the cell type.

## Results and discussion

### Alpha track structure and clustered damage

**Figure 5:**
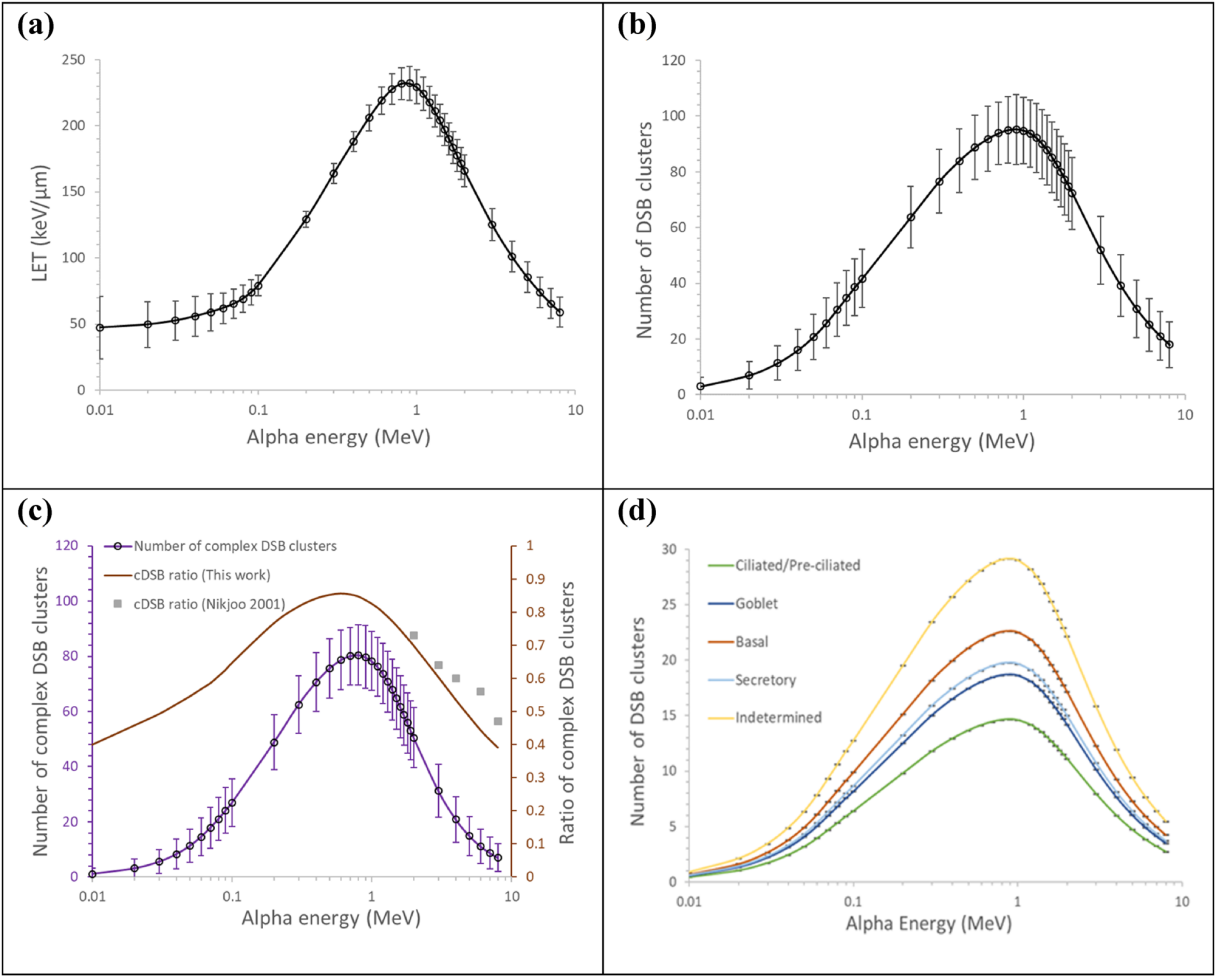
Results from Geant4-DNA simulation of energy deposition events in water medium followed by the DNA damage clustering over a 1 μm-thick volume segment: (**a**) LET, (**b**) the number of DSB clusters, (**c**) the number of complex DSB clusters and its ratio out of the total DSB clusters, and (**d**) the number of DSB clusters after applying the cell-specific volume fraction $p_{F}$ to the average values in (**b**). Each data point was obtained from the simulation of $10^{4}$ alpha particles. Each bar in (**a**–**c**) indicated 1.96 standard deviation to imply 95% of confidence interval of the estimate. Each bar in (**d**) indicates one standard deviation of the mean value.

Alpha particle was traced in a unit cylindrical volume of 5 μm in radius and 1 μm in thickness by using the Geant4-DNA. The track structure of a slowing down alpha particle in the unit volume was recorded in terms of LET, distribution and yields of energy depositions, DSB clusters, and complex DSBs. Figure 5a–c show the statistical variations in LET, the number of DSB clusters, and the number of complex DSBs, respectively, induced by alpha particles that started with an initial energy of 8 MeV and slowing down along the track. Each data point was obtained from $10^{4}$ histories. LET, DNA damage yield, and the damage complexity consistently increased as the alpha particle slowed down, peaked at around 1 MeV, and then decreased.

At energies below 60 keV, where the mean ranges of alpha particles were less than 1 μm, LET was recorded with greater variation due to energy loss straggling, as shown in Fig. 5a. The DSB yields decreased more slowly than LET after the peak. Even though the total energy loss by an alpha particle over 1 μm was reduced, the secondary electrons might have caused energy depositions in a more compact distribution. Our results showed good agreement with the data from Friedland et al.^28^ and Nikjoo et al.^31^ in the DNA damage yield and the ratio of complex DSBs among the total DSB clusters, respectively. Nikjoo et al.^31^ performed biophysical simulations of direct and indirect actions of particles on DNA. The nucleus sizes and the corresponding volume fraction of chromatin fiber $(p_{F} )$ in the nuclei of different lung epithelium cell types were reflected in the calculation of total number of DSBs to result in Fig. 5d. When the cell is large, the volume ratio of DNA substances in nucleus is reduced, and thus, the chance of DNA damage is low. Data in Fig. 5 established a library of statistically varied track history of an alpha particle carrying an energy of 8 MeV and less.

Our track simulation data indicated that the deposited energy and DSB clusters were concentrated in small radial distances from the main tracks of the slowing down alpha particles. With 8 MeV alpha particles, 96% of the deposited energy and 98% of the DSB clusters were located within a 100 nm radial distance from the main tracks (Supplement S.1). As alpha particles slowed down, the energy deposition within the 100 nm radial distance increased. This observation validated our SNU-CDS algorithm, which used the unit volume limited within 5 μm in radial distance to simulate damage in the cell nucleus. Previous Monte Carlo simulations suggested that the complex DSBs and their proximity are responsible for chromosome aberrations^59^ and that the ratio of mis-repaired DSBs increases with increasing proximity to each other^60^.

### Cellular damage due to a single alpha track

**Figure 6:**
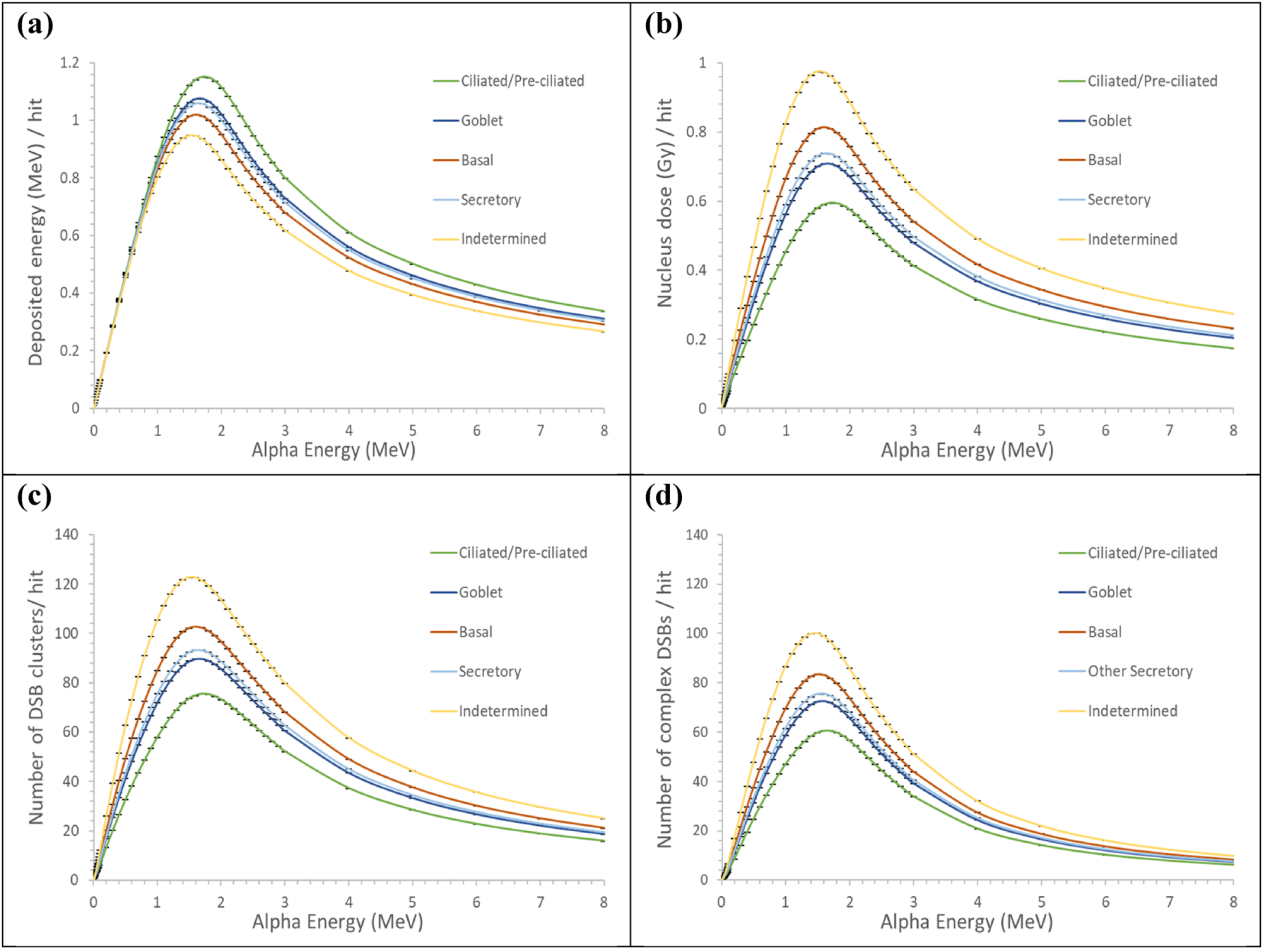
The average yields per nucleus hit by an alpha particle carrying different energies for different cell types in the lung tissue: (**a**) nucleus energy deposition, (**b**) nucleus dose, (**c**) number of DSB clusters, and (**d**) number of complex DSBs. Mean values were estimated using SNU-CDS through integration over the possible directions of an alpha particle upon entering. Each bar indicates one standard deviation of the mean value.

The alpha particles emitted from radon progeny residing in the luminal surface slowed down while traversing the mucous layer and the tissue segment. The alpha particles emitted from radon progeny residing in the mucous layer slowed down while traversing the mucous and serous layers and the tissue segment. Cellular damage caused by an alpha particle traversing the nucleus varied depending on the energy of the alpha particle upon entering the nucleus and the track length inside. The deep-sited cells would be hit by slowing down alpha particles. Track length inside the nucleus was maximum when the alpha particle was directed toward the center of the nucleus, and it decreased when the direction diverted from the center. Figure 6 summarizes the energy deposition in the cell nucleus, nucleus dose, and numbers of DSB clusters and complex DSBs, that are expected from a single alpha particle traversing the nucleus. Each cell type was modeled as spheres with the volumes listed in Table 1. The estimates were obtained by integrating the values over the possible directions of an alpha particle upon entering. The difference among cell types is attributed to the cell type-specific volume fractions of chromatin fiber in the nucleus $p_{F}$. The percentage of complex DSBs among all DBS clusters ranged from approximately 40% at the highest and lowest energies of the simulated alpha particles up to 82% at energies of approximately 1.2 MeV, as shown in Fig. 6c,d).

### Cellular damage in bronchial epithelium due to radon progeny

**Figure 7:**
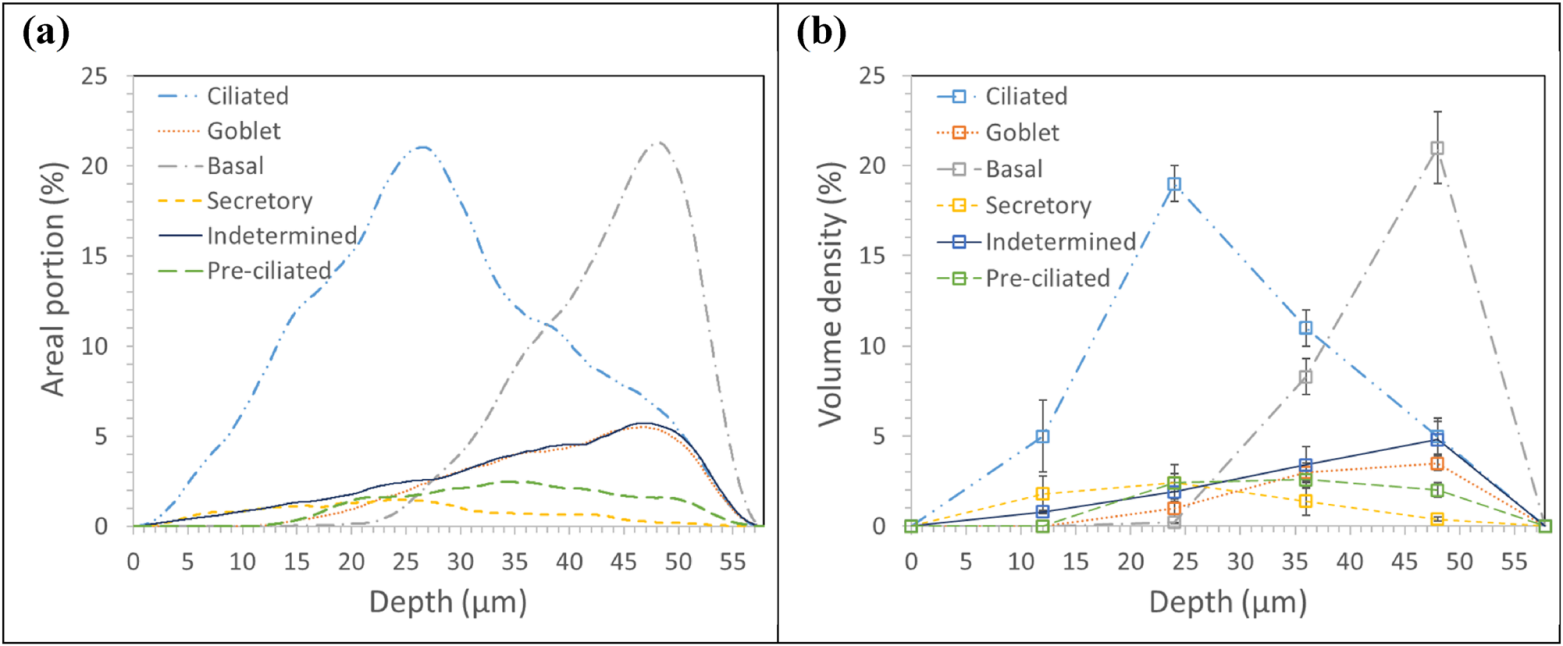
Areal portions of six cell types in the bronchial epithelium as functions of depth from the luminal surface: (**a**) estimates in this study at every 0.02 μm depth of the 57.8 μm-thick tissue segment linked between neighboring data points and (**b**) measurements by Mercer et al.^54^ at six discrete depths of the bronchial epithelium linearly interpolated between neighboring data points.

Figure 7a presents the areal portions of six cell types constituting the bronchial epithelium measured at every 0.02 μm depth of the 57.8 μm-thick tissue segment. In Fig. 7b, the data points in Table 2 are simply connected. The areal fractions evaluated along the depth of tissue were comparable with the reference functions^54^.

**Figure 8:**
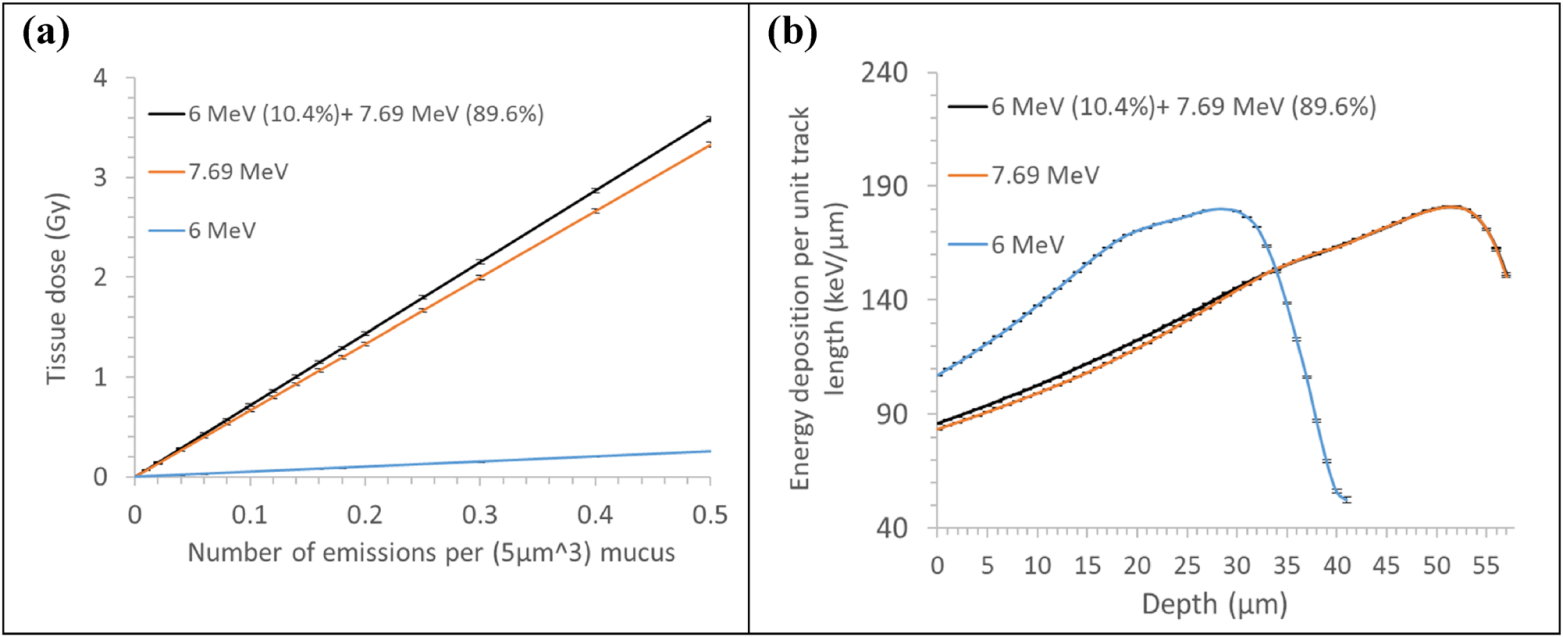
Simulation results: (**a**) dose of bronchial epithelium attributed to both or each of ^218^Po and ^214^Po emitting varied numbers of alpha particles in a 5 ${\upmu m}^{3}$($1\,\upmu{\text{m}} \times 1\,\upmu{\text{m}} \times 5\,\upmu{\text{m}}$) section of the mucous layer and (**b**) average energy depositions by alpha particles per unit length that vary in the depth in the tissue segment. Each bar indicates one standard deviation of the mean value.

Figure 8a presents the tissue dose, which varies with the depth from the luminal surface as a function of the total alpha-emission density from Po-218 (10.4%) and Po-214 (89.6%) radon progenies. Tissue dose increased linearly with the alpha-emission density by 7.17 Gy per unit alpha-emission density increase. The contributions of ${}_{ }^{214} {\text{Po}}$ and ${}_{ }^{218} {\text{Po}}$ to the tissue dose were 92.8% and 7.2%, respectively. The 6 MeV alpha particle emitted from Po-218 would lose more energy than the 7.69 MeV alpha particle emitted from Po-214 while traversing the 5 μm-thick mucosal and 6 μm-thick serous layers because of its higher LET. Hence, the contribution of 6 MeV alpha particles to the tissue dose further decreased. Figure 8b depicts the energy deposition per unit track length in the tissue segment. Alpha particles of 6 and 7.69 MeV in initial energy showed the highest energy deposition efficiency at 28 and 52 µm depths from the luminal surface, respectively. Consequently, the damage to the basal layer would be mostly due to the 7.69 MeV alpha particles originating from Po-214.

**Figure 9:**
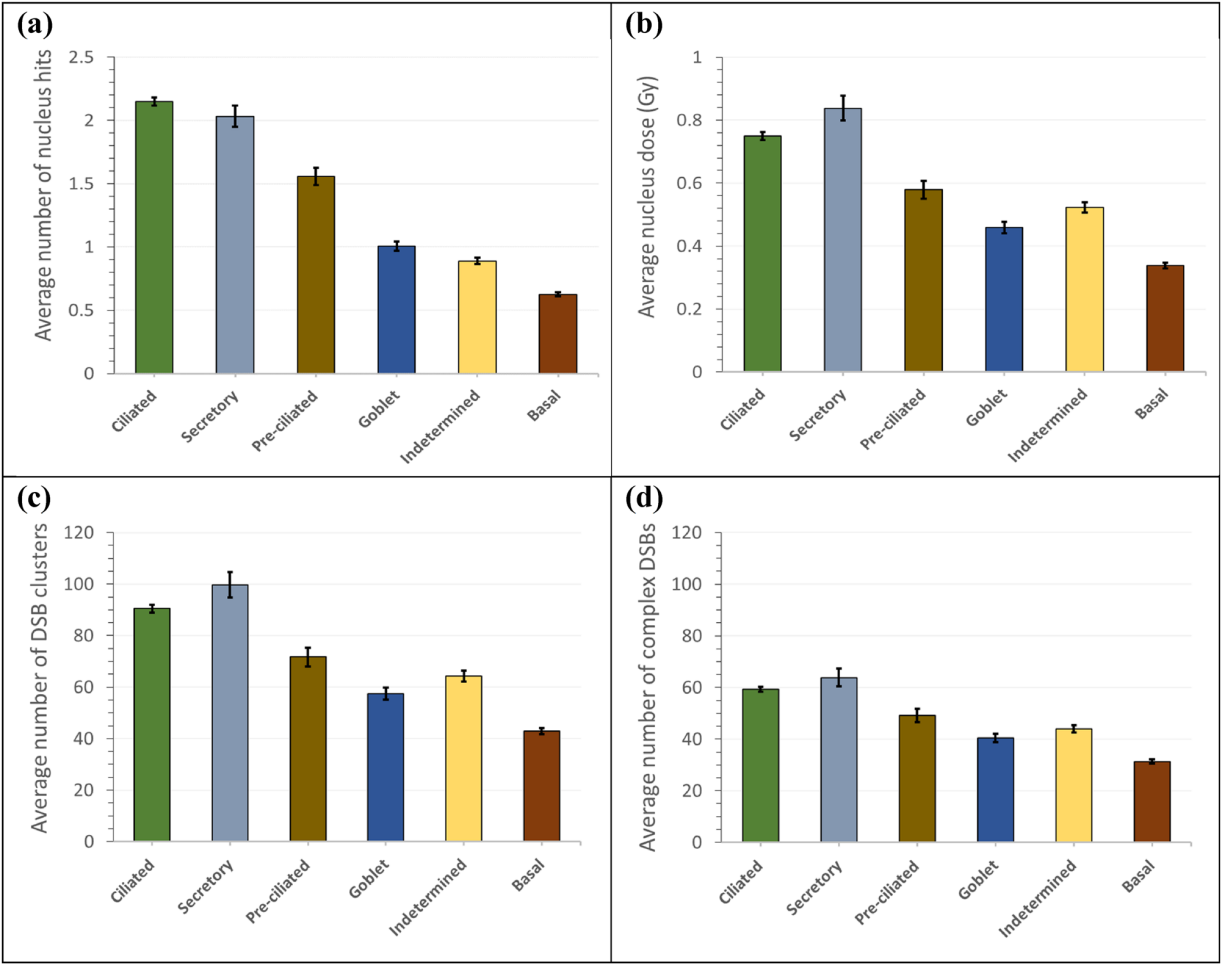
Average values of (**a**) number of nucleus hits, (**b**) nucleus dose, (**c**) number of DSB clusters, and (**d**) number of complex DSBs due to 0.1 alpha emission from radon progeny in a 5 ${\upmu m}^{3}$ mucosal layer. Each bar indicates one standard deviation of the mean value.

Six cell types were dispersed in the 57.8 μm-thick tissue segment. For each cell type, the chance of being hit was reduced at a deep site, but the average energy deposition to the cell increased upon being hit due to the high LET of low-energy alpha particle hitting the cell. Consequently, the nucleus dose per hit increased with increasing depth of the cell’s location in the tissue segment. High dose results in large numbers of DSB clusters and complex DSBs. With every hit by an alpha particle at a certain depth, the nucleus dose and the numbers of DSB clusters and complex DSBs became less in large cell nuclei compared with those in small cell nuclei. Figure 9 presents the average values assuming alpha emissions due to 0.1 disintegration of Po-218 and Po-214 radon progenies in a 5 ${\upmu m}^{3}$ mucus.

Ciliated nuclei had the highest chance of being hit, whereas basal nuclei had the lowest chance of being hit, as shown in Fig. 9a. Nucleus size (Table 1) and depth distribution in the tissue (Table 2) mattered for the chance of hit. Despite the highest chance of being hit, ciliated cell was the second highest in terms of nucleus dose and thus in the numbers of DSB clusters and complex DSBs. Secretory cell was the highest in terms of nucleus dose and DNA damage due to the high density of its DNA content in a smaller nucleus, as shown in Fig. 9b–d. Small and deep-located basal cells would be hit by the lowest chance and had the least DNA damage.

**Figure 10:**
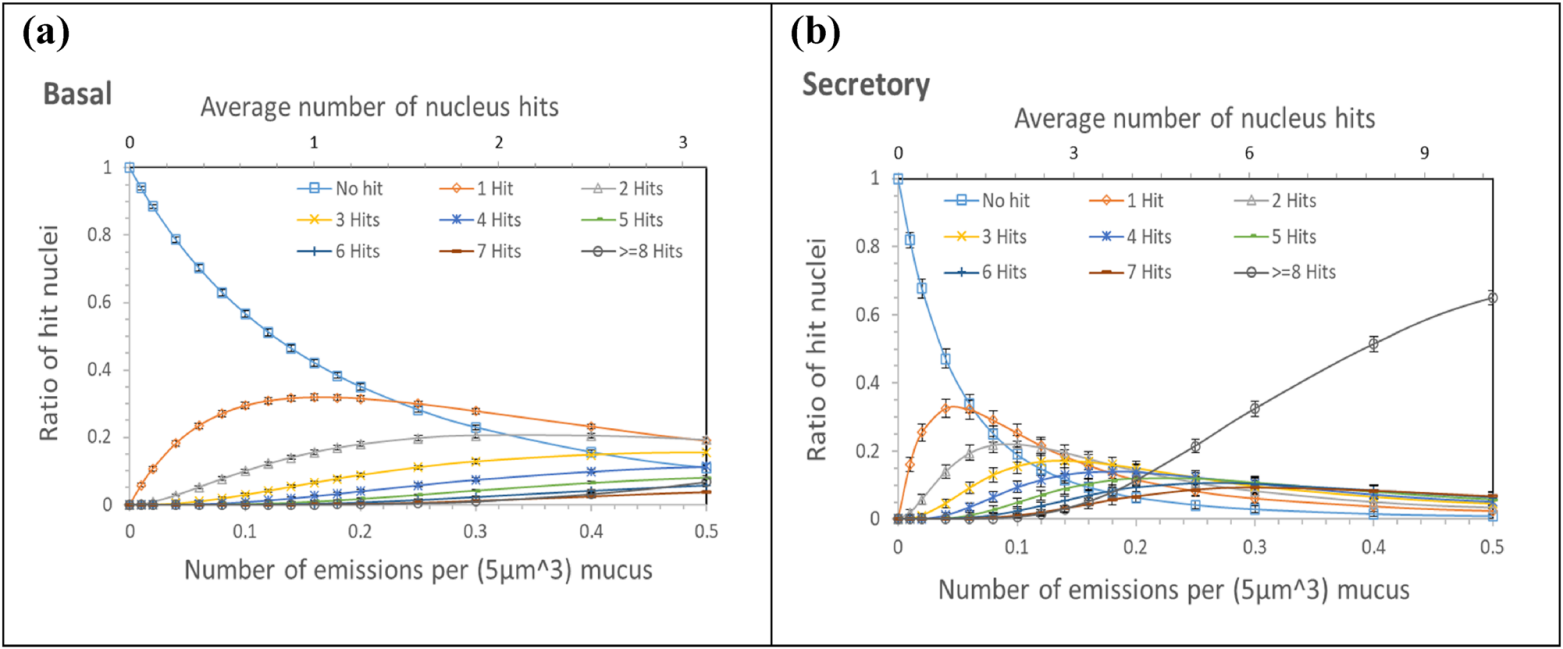
The ratio of nuclei that receive a specific number of hits due to varied numbers of alpha emissions from Po-218 and Po-214 radon progenies in a 5 ${\upmu m}^{3}$ mucosal layer for (**a**) basal and (**b**) secretory cells. Each bar indicates one standard deviation of the mean value.

The cell nuclei in the lung tissue would be hit multiple times with increased alpha-emission density. The mean number of hits can be calculated for varied alpha-emission densities by using the data in Fig. 9a. The hit probability differs among different cell types, and thus, the probability distribution of the number of hits also differs among cell types for a given alpha-emission density. Figure 10 presents the probability of the number of hits, which changes with the alpha-emission density, for basal and secretory cells. Due to alpha emissions at density of 0.5 per 5 ${\upmu m}^{3}$ mucus, ~ 10% of basal cells with 3.1 of average number of being hit have no chance of being hit and ~ 55% are hit less than 4 times (Fig. 10a). In contrast, over 65% of secretory cells with 10.2 of average number of being hit are hit by more than 8 times (Fig. 10b).

Previous studies reported that the atmospheric radon concentration in the New-Mexico mine was 5.7 WL^38^ and the hottest local dose in a 0.14 mm^2^ of the bronchial epithelium was estimated to be 5.29 Gy at an 8-h (working hours per day) exposure^14,20^. Working level (WL) is a measure of the concentration of radon progeny in $1{\text{ m}}^{3}$ of air with potential emission of $1.3 \times 10^{5} {\text{ MeV}}$ alpha energy $\left( {1{\text{ WL }} = { }20.8\,\upmu {\text{J m}}^{ - 3} { }} \right)$. Working level month (WLM) corresponds to the exposure for a working month (170 h) from breathing the air contaminated with 1 WL of radon progeny $\left( {1{\text{ WLM }} = { }3.54 \times 10^{ - 3} {\text{ J h m}}^{ - 3} } \right)$^23^. In our calculation, 5.29 Gy of tissue dose was due to 0.74 alpha emission in a 5 ${\upmu m}^{3}$ mucus. We calculated the WLM that would result in the density of alpha emissions for various tissue doses. In this way, we could provide the estimates for varying exposure conditions in terms of WLM as well as the density of alpha emissions: in the most exposed region of the lung epithelium, 0.1 and 0.5 alpha emission in a 5 ${\upmu m}^{3}$ mucus occurs at radon exposure of 0.036 and 0.18 WLM, respectively.

**Figure 11:**
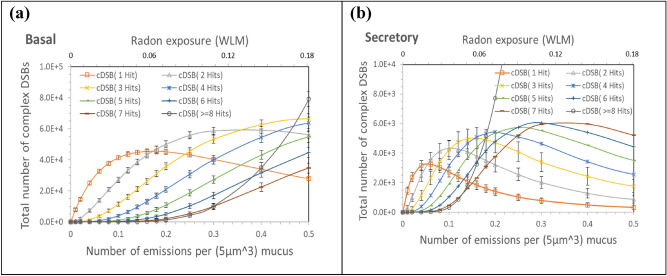
Total numbers of complex DSBs generated in all nuclei of the lung segment via different numbers of hits by alpha particles due to varied levels of radon exposure (up to 0.18 WLM): (**a**) basal and (**b**) secretory cells. The numbers of alpha emissions from a 5 µm^3^ mucus correspond to the levels of radon exposure, respectively. Each bar indicates one standard deviation of estimated mean value. Statistical uncertainty for secretory cells is high due to the lowest amount in the bronchial tissue. Estimated mean values of complex DSBs by more than four hits may lack statistical confidence.

Quantification of the complex DSBs from 8 h exposure of the progenitor cell nuclei results in realistic data. Considering that complex DNA damage is associated with slow repair^32–34,61^, complex DSBs from a hit by alpha particle would probably persist until additional hits occur in the nucleus in 8 h. The complex DSBs are the possible initiators of carcinogenic mutation^36^. Figure 9b shows that the highest average dose of around 0.85 Gy occurs to secretory cells due to 0.1 alpha emission from radon progeny in a 5 ${\upmu m}^{3}$ mucosal layer. We calculated the numbers of complex DSBs in the candidate progenitor cells, including the basal and secretory cells. Figure 11 presents the total numbers of complex DSBs generated via single and multiple hits on basal and secretory cells due to varied levels of radon exposure (up to 0.18 WLM). Basal cells had a larger portion of complex DSBs induced in the tissue segment than secretory cells, as shown in Fig. 11, due to more amount in the bronchial tissue (Table 1) and higher DSB yields per nucleus hit as shown in Fig. 6c,d. This finding is consistent with the results of Hofmann et al.^62^. Basal cells are located at deep sites in the tissue, and thus, hit by low-energy alpha particles. The efficiency of complex DSB generation per hit by low-energy alpha particles must be higher in basal cells (Fig. 11a) than in secretory cells (Fig. 11b). The majority of complex DSBs in basal cells could be generated via small numbers (2 or 3) of hits at the levels of radon exposure as high as 0.18 WLM due to low chance of hits at deep sites.

### Limitations and applicability

The depth distribution of target cells in the lung epithelium and the redistribution of radon progeny in the mucous layer through mucociliary action would greatly affect the simulation results. The data for depth distribution of target cells and distribution of radon progeny in the mucous layer used in our simulation were the best choices we could think of. Cell depth distributions in the lung epithelium of Mercer et al.^54^ are old data and may be updated. The kinetics of radon progeny in redistribution from deposition on the luminal surface can be further investigated. The calculational scheme developed in this study can be used to estimate DNA damage of cells in the lung tissue just by replacing the old data with the updated data. Updated data may lead to a different conclusion. Current calculational scheme was applied to several additional scenarios of radon progeny distribution in the mucous layer (Supplement S.2).

The current simulation scheme was developed by focusing on the nano-scaled track distribution of alpha particles without considering uncertain biological features, such as the sensitivity of different cells to radiation and varying radio-sensitivity during cell cycle. More practical estimation of the lung epithelium damage due to radon progeny is feasible when complete and accurate biological information becomes available.

## Conclusions

This study aimed to develop a scheme to simulate the nanometer-scaled DNA damage of cells in the lung tissue from radon exposure at a reasonable computational cost. We simulated alpha track structure on a nanometer scale by using the Geant4-DNA and incorporated the data of nanometer-scaled DNA damage in the micrometer-scaled track simulation, which was performed by using the Geant4 Livermore physics scheme. The performance of our new SNU-CDS algorithm, which links the nanometer- and micrometer-scaled simulations, generated data consistent with other studies.

The deep-sited basal cells in the lung epithelium had the largest portion of complex DSBs induced from exposure to alpha emissions of the radon progeny Po-218 and Po-214 distributed in the surface of the mucosal layer. The complex DSBs in basal cells would be attributed to 2 or 3 trajectories of the slowed-down alpha particles emitted at 7.96 MeV from Po-214.

## Statistical significance

Reliability of mean estimates was achieved by increasing the number of simulation histories. The chosen number of simulations resulted in mean estimates with fractional standard deviation less than 0.1.

## Supplementary Information

Supplementary Information.
