# Supplementary material for: Multi-scaled Monte Carlo calculation for radon-induced cellular damage in the bronchial airway epithelium
Source: Sci Rep. 2021 May 13;11:10230. doi: 10.1038/s41598-021-89689-0 (PMC8119983; doi:10.1038/s41598-021-89689-0)
Supplement: Supplementary file 1 — Supplementary Information. [file 41598_2021_89689_MOESM1_ESM.docx]

**Multi-scaled Monte Carlo calculation for radon-induced cellular damage in the bronchial airway epithelium**

**Ali Abu Shqair and Eun-Hee Kim^*^**

Seoul National University, Department of Nuclear Engineering, Seoul, 08826, Republic of Korea

*eunhee@snu.ac.kr

# **S.1 Radial distribution of energy depositions and DSBs**

Energy depositions and the number of double-strand breaks (DSB) clusters were recorded at different radial distances from the main tracks of the slowing-down alpha particles. Figure S1 shows the calculated cumulative distribution functions (CDF) at radial distances up to 1 μm from the main tracks.

| **a)**  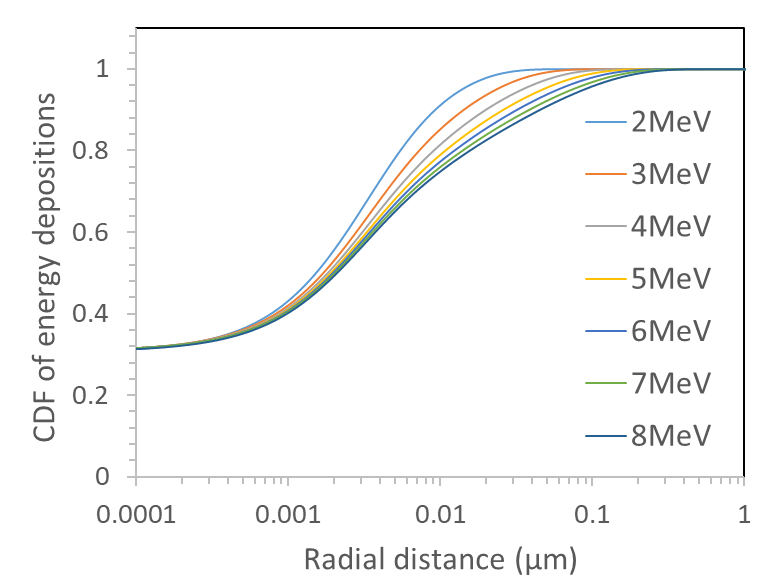 | **b)**  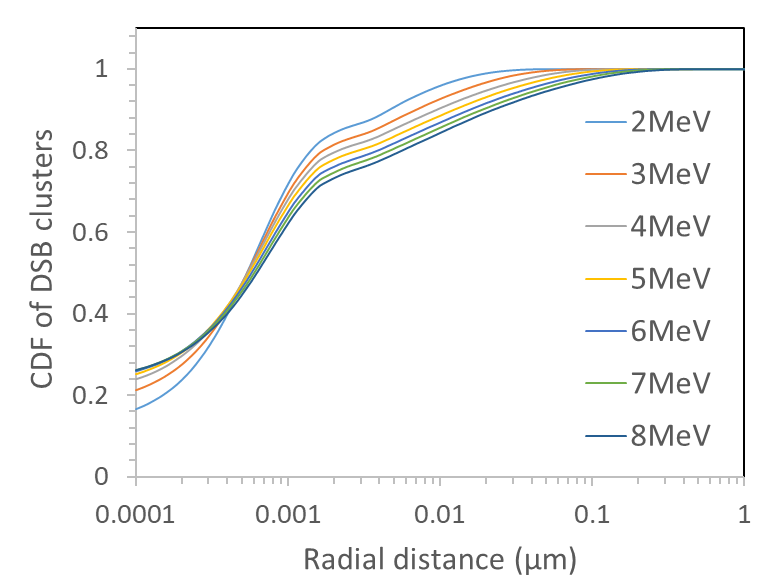 |
| --- | --- |
| Figure S1: The cumulative distribution functions (CDF) of (a) energy deposition and (b) number of DSB clusters as a function of the radial distance from the main alpha track. | |

# **S.2 Distribution of radon progeny in the mucous layer**

Simulations were performed with different initial settings for location and distribution of alpha decays in the mucous-serous layer. In the manuscript, alpha particles were assumed to originate from radon progenies uniformly distributed within the mucous gel layer (5 $\mu m$-thick), passing through the mucous and a clean serous layer (6 $\mu m$-thick) and reaching the epithelium cells. We tested our simulation model in comparison with three other simulation models regarding alpha-decay distribution pattern. First, alpha particles originate from radon progenies uniformly distributed within the 11 $\mu m$-thick mucous-serous layer. Second, alpha particles are emitted from the top surface of the 11 $\mu m$-thick mucous-serous layer. Lastly, the thickness of the mucous-serous layer was reduced to 5$\mu m$ and alpha particles are emitted from radon progenies uniformly distributed within the mucous layer. Figure S2 shows estimates of the three additional scenarios as compared to the scenario adopted in the manuscript.

Compared to the model adopted in the manuscript, alpha particles emissions from the 11 $\mu m$-thick mucous-serous layer resulted in the nucleus dose of basal and secretory cells increased by about 24% and 9%, respectively. The nucleus dose of basal and secretory cells were reduced by about 18% and 8%, respectively, when alpha particles were emitted from the surface of 11 $\mu m$-thick mucous-serous layer. Lastly, the nucleus dose of basal and secretory cells were increased by about 48% and 18%, respectively, when alpha particles emitted from the 5 $\mu m$-thick mucous layer.

| **a)**  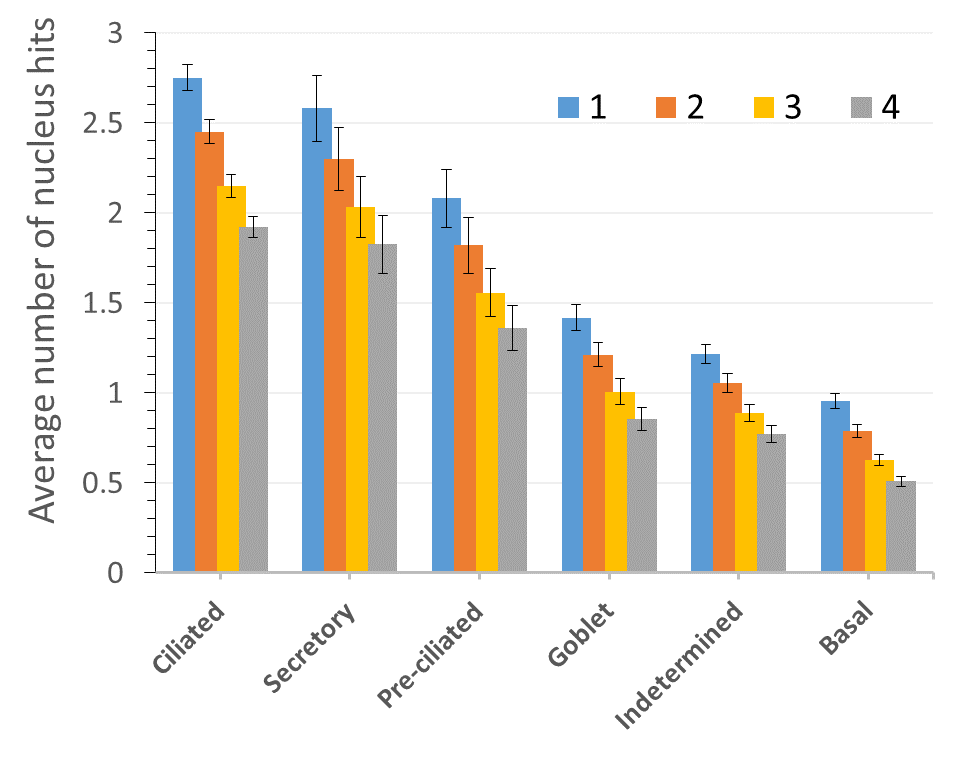 | **b)**  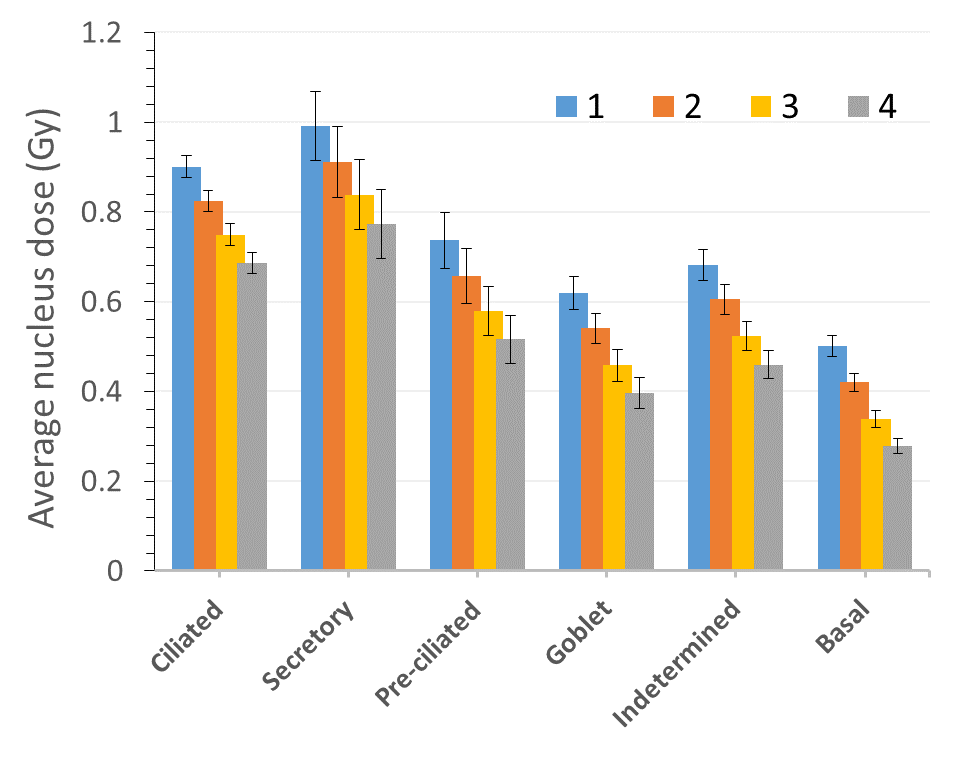 |
| --- | --- |
| **c)**  **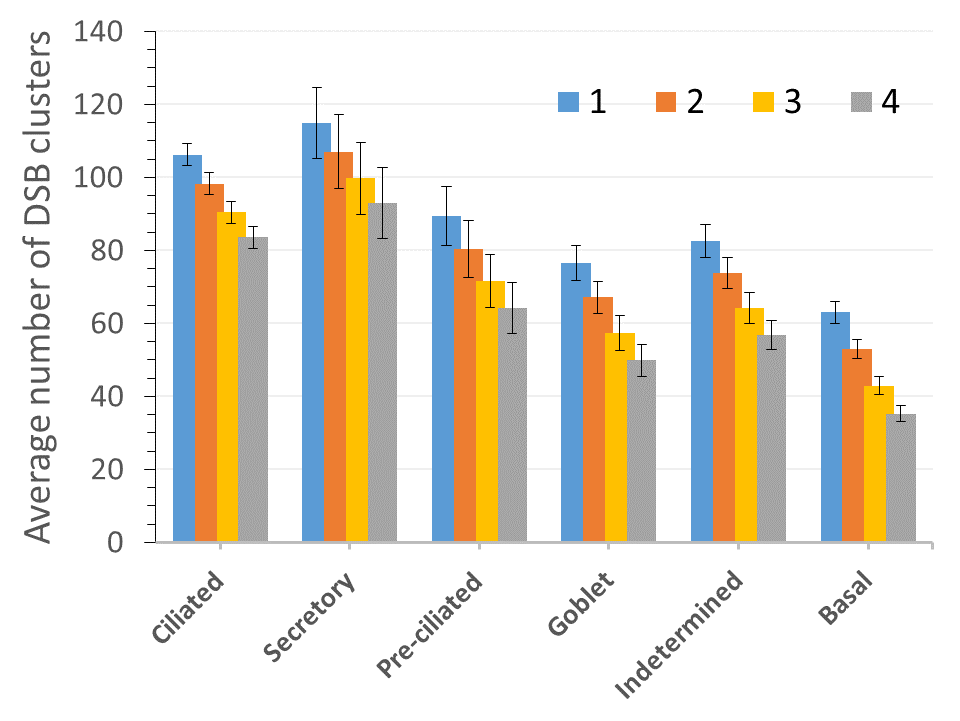** | **d)**  **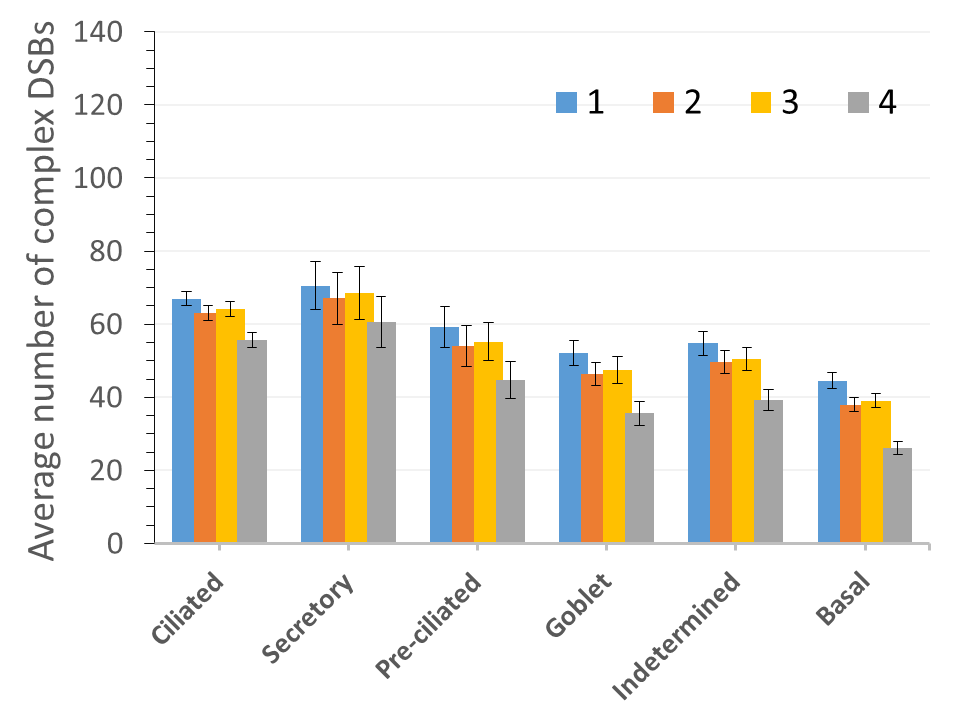** |
| Figure S2: Average values of (a) number of nucleus hits, (b) nucleus dose, (c) number of DSB clusters, and (d) number of complex DSBs due to 0.1 disintegration/μm^2^ of radon progenies in the surface of the mucosal layer. Four different cases of alpha-decay distributions are presented in order of resulting in nucleus doses from maximum (1) to minimum (4):   1. uniform within a 5$\mu m$-thick mucous layer, 2. uniform within a 11$\mu m$-thick mucous-serous layer, 3. uniform within a 5$\mu m$-thick mucous gel layer over a clean (no decays) 6 $\mu m$-thick serous layer, and 4. uniform on the surface of a 11$\mu m$-thick mucous-serous layer. | |
|  | |

|  |
| --- |
